# Autologous bone marrow transplantation in non-Hodgkin lymphoma patients following different conditioning regimens: an 11-year single-center quasi-experimental study

**DOI:** 10.1007/s44313-026-00131-8

**Published:** 2026-04-09

**Authors:** Sahar Tavakoli Shiraji, Mohammad Biglari, Soroush Rad, Maryam Barkhordar, Hossein Kamranzadeh Foumani, Ghasem Janbabaei, Seied Asadollah Mousavi, Mohammad Vaezi, Kamran Mohammadi, Ardeshir Ghavamzadeh

**Affiliations:** 1 Research Institute for Oncology, Hematology and Cell Therapy, Tehran University of Medical Sciences, Tehran, Iran; 2 Hematologic Malignancies Research Center, Tehran University of Medical Sciences, Tehran, Iran; 3 Verspeeten Family Cancer Center, London Health Sciences Research Institute, University of Western Ontario, London, ON Canada; 4 Hematology, Oncology and Stem Cell Transplantation Research Center, Tehran University of Medical Sciences, Tehran, Iran; 5 Cell Therapy and Hematopoietic Stem Cell Transplantation Research Center, Tehran University of Medical Sciences, Tehran, Iran; 6 School of Medicine, Tehran University of Medical Sciences, Tehran, Iran

**Keywords:** Autologous stem cell transplantation, Carboplatin, Conditioning, Non-Hodgkin lymphoma, Survival

## Abstract

**Purpose:**

Non-Hodgkin lymphoma (NHL) constitutes 90% of all lymphomas and accounts for 2.8% of new cancer cases and 2.6% of cancer-related deaths in 2020. Hematopoietic stem cell transplantation remains the standard treatment for refractory or relapsed NHL. Due to the shortage of carmustine, this study aimed to compare the outcomes of patients undergoing autologous stem cell transplantation (ASCT) with conditioning regimens that either include or exclude this drug over an 11-year period.

**Methods:**

This retrospective study included a cohort of 240 patients with NHL who underwent ASCT with an EAM conditioning regimen, both with and without carboplatin. Following informed consent, clinical data from patients who underwent transplantation between March 2006 and May 2017 were collected. Patients were followed to assess overall survival (OS) and disease-free survival (DFS) as primary endpoints.

**Results:**

No significant differences were observed between the survival outcomes of the two groups with differing conditioning regimens (*P* = 0.27 for OS; *P* = 0.49 for DFS). The 3-year and 5-year OS rates were 81% and 74.8%, respectively, with a median follow-up of 74 months. The 3-year and 5-year DFS rates were 66.6% and 62.1%, respectively. OS and DFS were significantly higher in patients with B-cell lymphomas compared to those with T-cell lymphoma (*P* < 0.01).

**Conclusion:**

Our real-world data indicate that the addition of carboplatin to the EAM conditioning regimen does not significantly affect survival outcomes for patients with NHL undergoing ASCT.

## Introduction

According to the World Health Organization classification, non-Hodgkin lymphoma (NHL) represents a heterogeneous group of malignancies that can originate from either B or T lymphocytes at various stages of maturity [1]. NHL accounts for 90% of all lymphoma cases [2]. In 2020, approximately 540,000 new cases of NHL were reported, representing 2.8% of all newly diagnosed cancers. Additionally, NHL was responsible for about 260,000 deaths in 2020, accounting for 2.6% of cancer-related deaths. The incidence of NHL has been increasing, particularly in developed countries, although the underlying reasons remain unclear [3]. According to data from the Surveillance, Epidemiology, and End Results program, the overall 5-year survival rate for NHL is approximately 74% [4].

NHL treatment varies by lymphoma subtype and disease characteristics, including stage and grade. The most common first-line treatments for NHL are chemotherapy, immunotherapy, radiotherapy, or combinations of these modalities [5]. Despite significant advancements in NHL management, many patients fail to achieve complete remission or experience early relapse [6]. For relapsed or high-risk NHL, hematopoietic stem cell transplantation (HSCT) remains a key therapeutic approach [7–9].

HSCT has long been the treatment of choice for high-grade and relapsed NHL. Current guidelines from the American Society of Clinical Oncology (ASCO), the European Hematology Association, and the National Comprehensive Cancer Network recognize chemotherapy followed by autologous HSCT as the standard treatment for refractory/relapsed NHL [10–12]. Autologous HSCT (ASCT) improves overall survival (OS) in patients with NHL. However, newer approaches, such as chemoimmunotherapy, have also enhanced both overall and disease-free survival (DFS). Immunomodulators and small molecule inhibitors, such as ibrutinib (a Bruton's tyrosine kinase [BTK] inhibitor) and idelalisib (a phosphoinositide 3-kinase [PI3K] inhibitor), have reshaped the therapeutic landscape, redefining the role of ASCT in clinical management [13–15].

The most important factor in determining NHL outcomes prior to ASCT is chemosensitivity. In chemosensitive disease, with an ASCT mortality rate of less than 5%, a cure rate of approximately 50% is expected [16–18]. Other prognostic factors include the histologic features of NHL, aggressiveness and stage of the disease, serum LDH levels, and the number of prior treatments [19–21]. Several high-dose therapy (HDT) regimens, which vary greatly in toxicity and effectiveness, have been used for ASCT preparation. The carmustine (BCNU), etoposide, cytarabine, and melphalan (BEAM) regimen is the most widely accepted and well-studied protocol. However, limited access to BCNU and its high cost in many countries have led many institutions to omit it or replace it with alternative agents [22].

Reports have highlighted the use of the EAM regimen (BEAM without BCNU), showing promising efficacy comparable to the BEAM protocol [23–25]. Here, we present our real-world survival data comparing the EAM regimen to CEAM (addition of carboplatin) due to the BCNU shortage in our country. Additionally, we explore the impact of disease characteristics and treatment history on prognosis.

## Materials and methods

This retrospective study was conducted at the Research Institute of Oncology, Hematology, and Cell Therapy, affiliated with Tehran University of Medical Sciences (TUMS), Tehran, Iran. Adult patients with pathologically confirmed NHL who underwent autologous HSCT between March 2006 and May 2017 were included. The following protocols were administered based on the specified timeframes. From 2006 to 2014, the EAM regimen was used, which included etoposide 600 mg/m^2^ on days −3 and −2; cytarabine 1250 mg/m^2^ bid on days −3 and −2; and melphalan 140 mg/m^2^ on day −1, all as intravenous infusions. From 2014 to 2017, carboplatin was added to the regimen at 200 mg/m^2^ on day −4 (CEAM). Peripheral blood and granulocyte colony-stimulating factor (GCSF)-primed, unmanipulated autologous hematopoietic stem cells were infused on day 0.

Inclusion criteria required patients to be over 18 years old and to have achieved the maximum response at the time of transplant. Informed consent was obtained from all patients. Exclusion criteria included a history of previous malignancy or bone marrow transplant, pregnancy, breastfeeding, or incomplete data.

All consecutive patients meeting the inclusion criteria were enrolled, and relevant demographic and clinical data were recorded. Standard supportive care measures following transplant included GCSF support, blood product transfusion, and antimicrobial prophylaxis at the physician’s discretion. The last follow-up was conducted in January 2023. Primary endpoints of the study were OS and DFS. Survival status was compared between the two timeframes: the EAM regimen (2006–2014) and the CEAM regimen (2014–2017). Additionally, non-relapse mortality (NRM) within the defined time period was assessed as a secondary endpoint. Neutrophil and platelet engraftment were defined as the first consecutive three days with an absolute neutrophil count greater than 0.5 × 10^9^/L and platelet count greater than 20 × 10^9^/L, along with independence from blood product transfusion for a minimum of 7 days, respectively.

The following definitions were used in the study protocol. NRM was defined as all deaths occurring without prior disease relapse or progression. Disease progression after transplantation, diagnosed by imaging or biopsy, was considered a relapse. The event for OS was death from any cause after transplant, and the event for progression-free survival (PFS) consisted of death from any cause or relapse. Staging was performed according to the American Joint Committee on Cancer, 8th edition [26]. Each course of chemotherapy using a different regimen for episodes of disease relapse before transplantation was considered a line of treatment.

To analyze overall and DFS, log-rank analysis and Kaplan–Meier curves were used. For survival predictors, Cox regression analysis was performed. Cumulative incidence curves and Gray's method were employed to analyze and compare relapse rates, NRM, and neutrophil and platelet engraftment. All data were analyzed using IBM SPSS software version 26 (IBM Corp, Armonk, NY, USA) and MedCalc version 20 (MedCalc Software, Ostend, Belgium). A p-value of less than 0.05 was considered significant. Approval was obtained from the ethics committee of TUMS, and the procedures adhered to the tenets of the Declaration of Helsinki.

## Results

A total of 240 patients diagnosed with NHL underwent ASCT during the study period. The median age was 37.3 years, with the majority being male (*n* = 151, 62.9%). Most patients had B-cell NHL (83.1%), with Diffuse Large B-cell lymphoma (DLBL) as the most common subtype (49.6%); 17% had T-cell lymphoma. Four-fifths had received at least two chemotherapy lines before ASCT, with an average of 2.2 lines, and all had at least one line of chemotherapy. Clinical and pathological features are detailed in Table 1.

**Table 1:** Clinicopathological data of patients

Characteristic	Category	Value, n (%)
**Age—median (years)** **range (years)**		37.3 8–70
**Sex**	Male Female	151 (62.9) 89 (37.1)
**NHL subtype**	DLBL Other B-cell lymphoma Mantle cell lymphoma High-grade B-cell lymphoma, NOS T-cell-rich B-cell lymphoma Follicular lymphoma Marginal zone lymphoma Burkitt lymphoma Primary CNS lymphoma T-cell lymphoma Peripheral T-cell lymphoma Anaplastic Large T-cell lymphoma Angioimmunoblastic T-cell lymphoma Panniculitis-like T-cell lymphoma Lymphoblastic lymphoma Unknown	119 (49.6) 50 (20.8) 10 10 13 9 4 3 1 41 (17.1) 22 16 2 1 26 (10.8) 4 (1.7)
**Stage at diagnosis**	I II III IV Unknown	41 (17.1) 92 (38.3) 53 (22.1) 43 (17.9) 11 (4.6)
**Lines of chemotherapy**	1 2 3 4 ≥ 4	48 (20) 128 (53.3) 41 (17.1) 16 (6.7) 7 (2.9)
**Disease status prior to ASCT**	Remission Residual disease	171 (71.2) 69 (28.8)
**Conditioning regimen**	EAM CEAM	165 (68.7) 75 (31.3)

*ASCT* Autologous stem cell transplantation, *EAM* Etoposide, Cytarabine, Melphalan, *CEAM* Carboplatin, Etoposide, Cytarabine, Melphalan, *CNS* Central nervous system, *DLBL* Diffuse large B-cell lymphoma, *NHL* Non-Hodgkin lymphoma, *NOS* Not otherwise specified

Chemotherapy regimens prior to ASCT were selected based on the specific lymphoma subtype. The standard chemotherapy regimen for T-cell and most B-cell lymphomas in the frontline setting is the CHOP regimen (cyclophosphamide, doxorubicin, vincristine, prednisolone). For lymphoblastic lymphoma, a higher-dose Mega-CHOP regimen was used as initial treatment. However, a highly intensive combination regimen consisting of cyclophosphamide, vincristine, doxorubicin, methotrexate, ifosfamide, etoposide, and cytarabine (CODOX/M-IVAC) was employed for all Burkitt lymphoma patients. Rituximab was used in cases of CD20-positive disease. Radiation therapy was part of the treatment in 12 patients prior to ASCT.

**Fig. 1:**
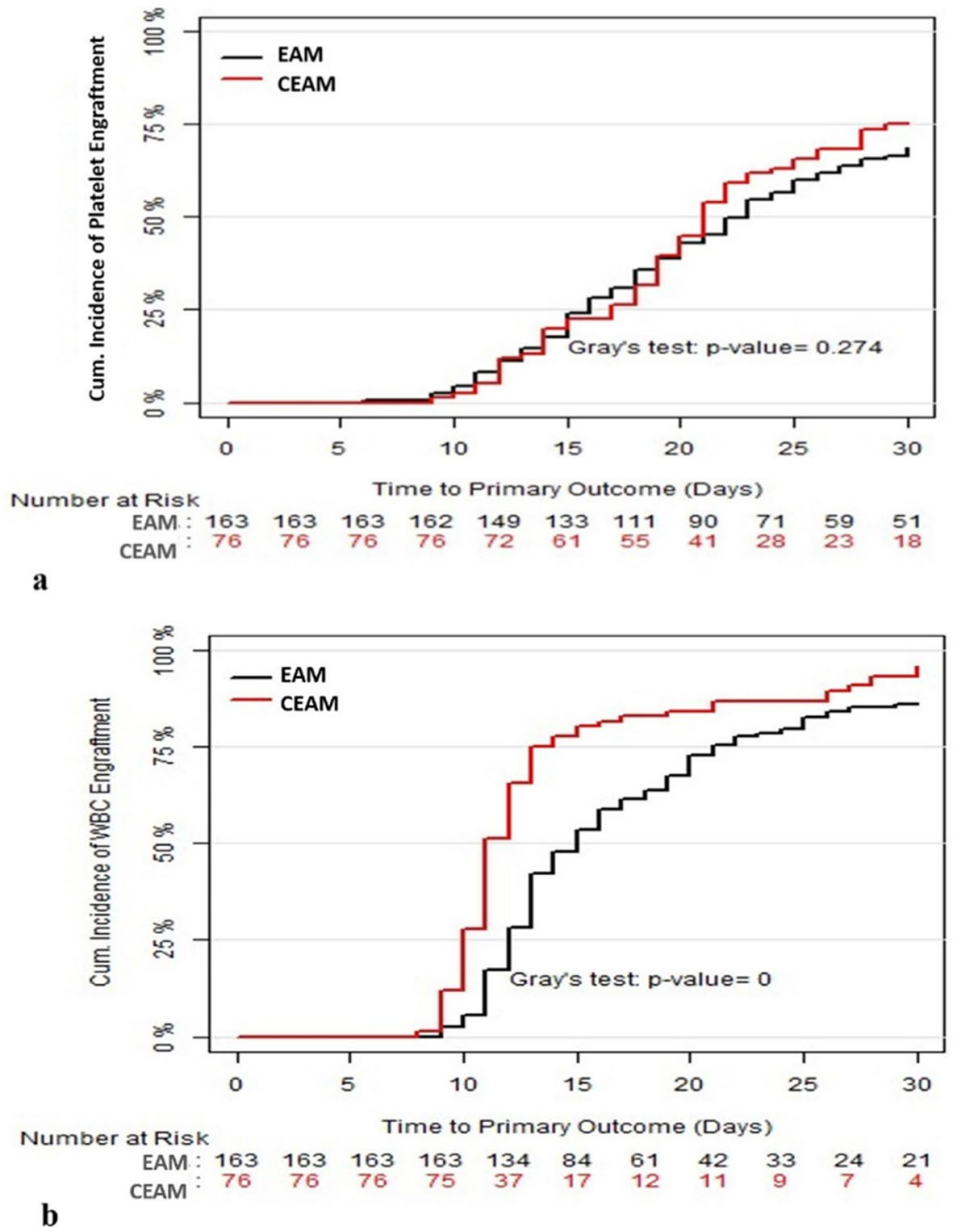
The cumulative incidence curve for **a** platelet and **b** neutrophil engraftment

The conditioning regimen in nearly two-thirds of the patients consisted of triple therapy with EAM, whereas the remaining third received the CEAM regimen prior to transplant. The mean time interval from diagnosis to ASCT was 31.1 ± 20.6 months. Only 15 patients required the CXCR4 inhibitor plerixafor prior to stem cell collection, and the average amount of CD34 + cells infused was 2.7 ± 1.0 × 10^9^/kg. The most common transplant-related toxicities were neutropenia and anemia (100%), followed by infectious complications (82%) and mucositis (78%). Median platelet and neutrophil engraftment times were 20.9 days and 13.3 days, respectively. Patients remained in the hospital for a median of 24.3 days (range 19–29). Table 2 summarizes the transplant's immediate outcome data. The median time to neutrophil recovery was significantly longer in patients receiving the CEAM regimen compared to EAM (11.1 vs. 14.9 days, *P* < 0.01), while platelet recovery time showed no difference. The cumulative incidence curve for platelet and neutrophil engraftment is shown in Fig. 1.

**Table 2:** Major immediate outcomes of ASCT

Outcome	Category	Value, n (%)
Platelet engraftment time in days, median (range)		20.9 (17–26)
Neutrophil engraftment time in days, median (range)		13.3 (10–18)
Mucositis	Grade I/II Grade III/IV	173 (72.1) 48 (20)
Neutropenia	Grade I/II Grade III/IV	138 (57.5) 102 (42.5)
Anemia	Grade I/II Grade III/IV	175 (72.9) 65 (27.1)
Infectious complication	Pneumonia Diarrhea Positive blood culture Others	45 (18.7) 39 (16.2) 22 (9.2) 14 (5.8)

*ASCT* Autologous stem cell transplantation

The CEAM conditioning regimen caused neutropenia more frequently than the EAM regimen (74.7% vs. 27.8%, *P* = 0.01). Additionally, grade III/IV mucositis was more commonly observed with the CEAM regimen, though this difference was not significant (42.9% vs. 23.2%, *P* = 0.063). There was no significant difference in the incidence of diarrhea, bacteremia, and anemia between the two conditioning protocols (Table 3).

**Table 3:** Grade III/IV toxicity data for EAM or CEAM conditioning

	**EAM (*n* = 165)**	**CEAM (*n* = 75)**	***P* value**
Neutropenia	46 (27.8%)	56 (74.7%)	0.01
Anemia	45 (27.3%)	20 (26.7%)	0.86
Mucositis	20 (12%)	28 (37.3%)	0.07
Infectious complications Pneumonia Bacteremia Diarrhea	29 (17.6%) 15 (9.1%) 28 (16.9%)	16 (21.3%) 7 (9.3%) 11 (14.7%)	0.76 0.61 0.45

The median follow-up time for patients receiving the EAM regimen was 89 months, and for the CEAM regimen, it was 57 months, with the overall median follow-up being 74 months. The 3-year and 5-year OS rates were 81.0% and 74.8%, respectively, and the 3-year and 5-year DFS rates were 66.6% and 62.1%, respectively. The major factor affecting both OS and DFS was the pathologic type of the disease, with other B-cell lymphomas showing the best survival (Table 4). Data indicated that stage I disease had the best 5-year DFS and OS, although this finding was not significant according to the log-rank test (ꭓ^2^ = 6.25, *P* = 0.1 for OS; ꭓ^2^ = 3.62, *P* = 0.3 for DFS).

**Table 4:** Survival data in patients with NHL undergoing ASCT

Variable	Category	3-y DFS	5-y DFS	*P* value ^*^	3-y OS	5-y OS	*P* value ^*^
Overall		66.6	62.1		81.0	74.8	
Lymphoma subtype	DLBL Other B-cell lymphoma T-cell lymphoma Lymphoblastic lymphoma	72.4 75.6 47.2 56.5	68.8 69.3 41.2 56.5	< 0.01	85.0 89.7 71.1 62.6	78.6 84.9 65.2 62.6	0.03
Stage at diagnosis	I/II III/IV	67.5 62.3	64.2 56.4	0.28	83.5 68.6	77.6 67.5	0.08
Lines of chemotherapy	1 2 3 4	81.0 65.5 57.1 61.8	74.3 60.6 54.7 61.8	0.21	87.2 82.7 69.6 86.7	87.2 72.9 66.7 77.5	0.15
Conditioning regimen	EAM CEAM	65.0 70.0	60.7 64.6	0.49	80.5 82.2	72.4 80.3	0.27

*ASCT* Autologous stem cell transplantation, *DFS* Disease-free survival, *DLBL* Diffuse Large B-cell lymphoma, *EAM* Etoposide, Cytarabine, Melphalan, *CEAM* Carboplatin, Etoposide, Cytarabine, Melphalan, *NHL* Non-Hodgkin lymphoma, *OS* Overall survival

^*^P value is given for the 5-year survival measures

**Fig. 2:**
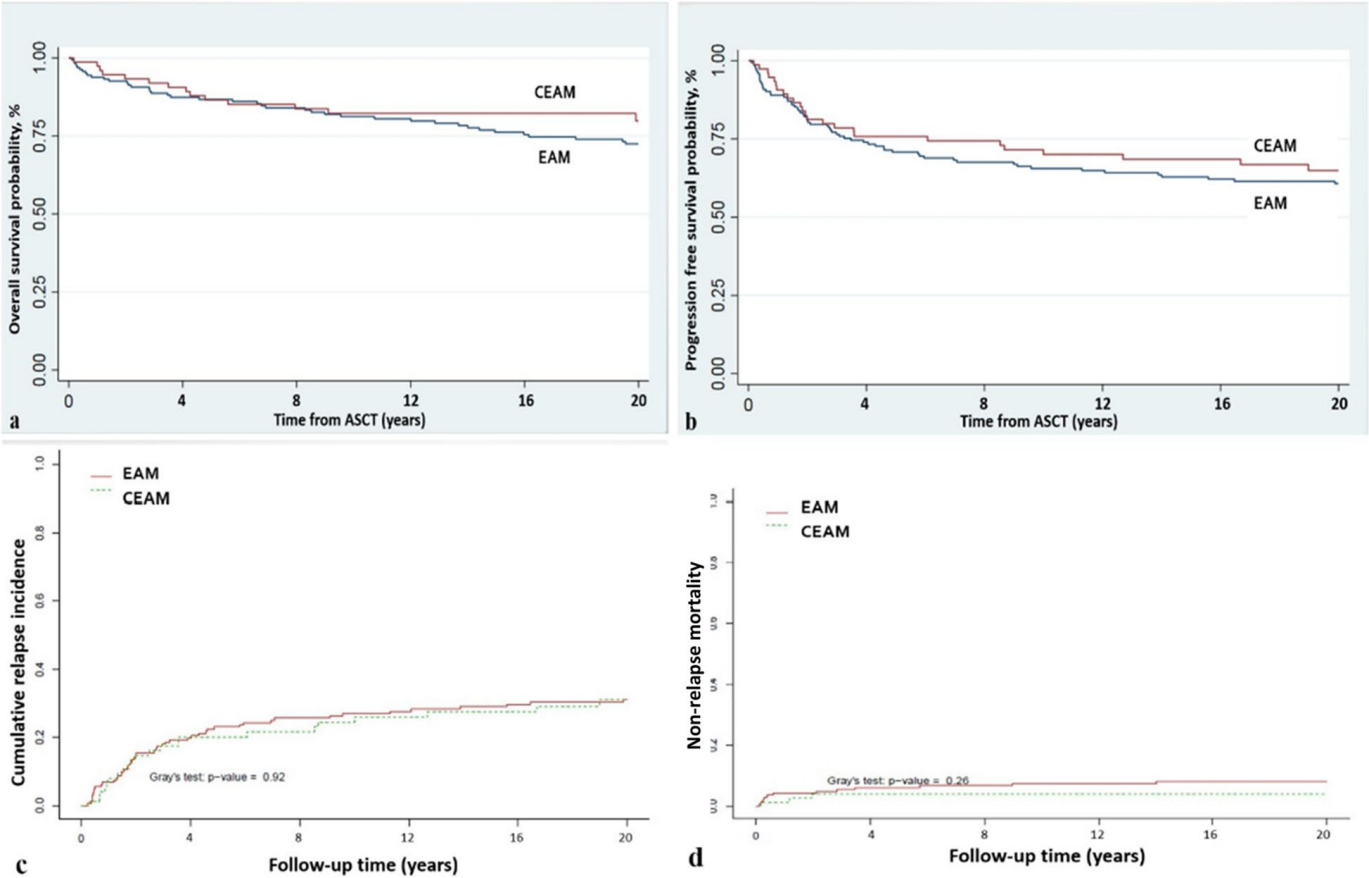
Outcomes of autologous stem cell transplantation (ASCT) stratified by conditioning regimen. **a** Overall survival, **b** Progression-free survival, **c** Relapse rate **d** Non-relapse mortality rate

No significant difference was found in the 5-year OS (72.4% [95% CI 64.8–90.7] vs. 80.3% [95% CI 73.8–95.7], *P* = 0.27) and 5-year DFS (60.7% [95% CI 52.7–76.0] vs. 64.6% [95% CI 52.7–67.1], *P* = 0.49) between the EAM and CEAM conditioning groups. Furthermore, there was no statistically meaningful difference between the EAM and CEAM regimens in the incidence of NRM and relapse (*P* = 0.26 for NRM and 0.92 for relapse) (Fig. 2).

Univariate Cox regression analysis showed that more than two lines of chemotherapy, disease stage at diagnosis, and T-cell pathology were significantly associated with shorter overall and disease-free survival. To obtain more accurate results and identify independent prognostic factors for ASCT outcomes, multivariate Cox regression was performed, which further confirmed that T-cell lymphoma and lower initial stage are negative predictive factors for patients with NHL outcomes. The choice of conditioning regimen was not found to affect survival in the Cox regression model (Table 5).

**Table 5:** Univariate and multivariate Cox regression survival analysis

Factors	Univariate				Multivariate			
	DFS		OS		DFS		OS	
	HR (95% CI)	*P* value	HR (95% CI)	*P* value	HR (95% CI)	*P* value	HR (95% CI)	*P* value
**Age**	1.02 (1–1.04)	0.03	1.02 (1–1.04)	0.06	1.02 (1–1.04)	0.04	1.02 (0.99–1.04)	0.11
**Time to transplant**	0.99 (0.98–1)	0.4	0.99 (0.98–1)	0.2	0.99 (0.98–1)	0.12	0.99 (0.97–1)	0.18
**Lymphoma subtype**	1.16 (0.95–1.43)	0.15	1.12 (0.89–1.41)	0.34	1.25 (1–1.57)	0.04	1.05 (0.79–1.39)	0.76
**Disease stage**	1.11 (0.88–1.41)	0.37	0.91 (0.7–1.18)	0.49	1.1 (0.86–1.41)	0.43	0.9 (0.68–1.19)	0.46
**Lines of chemotherapy**	1.17 (0.91–1.51)	0.23	1.13 (0.86–1.5)	0.38	1.2 (0.89–1.61)	0.23	1.14 (0.81–1.61)	0.45
**Conditioning regimen**	1.19 (.72–1.97)	0.48	0.71 (0.39–1.26)	0.24	1.24 (0.73–2.11)	0.42	0.67 (0.34–1.34)	0.26

*DFS* Disease-free survival, *DLBL* Diffuse Large B-cell lymphoma, *EAM* Etoposide, Cytarabine, Melphalan, *CEAM* Carboplatin, Etoposide, Cytarabine, Melphalan, *HR* Hazard ratio, *OS* Overall survival

## Discussion

NHL encompasses a diverse group of B-cell, T-cell, and natural killer cell neoplasms arising from various stages of maturity [27]. This disease is recognized as the fifth to ninth most common cause of cancer-related mortality worldwide [28]. High-dose chemotherapy (HDT) followed by ASCT remains a viable and promising therapeutic option for many patients with refractory or relapsed NHL. Nevertheless, no conditioning regimen has been established as definitive, and the choice largely depends on transplant centers.

Among the several preparation protocols for ASCT, the BEAM regimen (comprising carmustine, etoposide, cytarabine, and melphalan) has the most robust evidence supporting its use in both NHL and Hodgkin lymphoma [22, 23, 29–32]. Despite its popularity and effectiveness, limited access to carmustine and its high cost in many regions, including our country, hinders the widespread use of BEAM, prompting institutions to either omit or substitute it with alternative agents [23–25].

We retrospectively assessed the long-term outcomes of relapsed or refractory patients with NHL undergoing two conditioning regimens lacking carmustine (EAM and CEAM), due to its unavailability in our country. After a median follow-up of 74 months for 240 patients, the 3-year and 5-year OS rates were 81.0% and 74.8%, respectively. These results are consistent with previous studies using the BEAM regimen, which reported a 3-year OS ranging from 56 to 75% [33–35].

Although adding carboplatin to the EAM regimen resulted in a shorter interval to neutrophil engraftment, the conditioning regimen did not significantly impact survival, relapse rates, or NRM. The poorest outcomes were observed in T-cell lymphoma patients compared to those with DLBL and other B-cell lymphomas. The prognosis of patients with NHL was closely associated with disease staging.

The observed similarity in survival outcomes after the addition of carboplatin to EAM warrants further explanation. Our patients predominantly had chemosensitive disease, indicating a generally better response to chemotherapy agents. This suggests that drug resistance may have a lesser role in disease progression and that lymphoma-specific factors, such as disease burden and tumor molecular behavior, are more likely contributors. The addition of carboplatin to overcome cross-resistance in this scenario may not be helpful, particularly if patients experience late relapses, which may point to chemosensitive clones escaping treatment and contributing to progression. This highlights the importance of considering host factors, including hematopoietic reserve and tolerability, when selecting an appropriate conditioning regimen.

Numerous studies have evaluated a diverse array of HDTs prior to autologous transplantation in NHL. However, none of these studies comparing HDT protocols were randomized or prospective [30]. Research on patients with pulmonary issues prior to transplant has demonstrated the feasibility of removing carmustine from the BEAM regimen, although the outcome was inferior to the BEAM protocol (OS of 29 vs. 77 months, *P* = 0.03) [24]. Pangarsa et al. showed that EAM can be used in patients with Hodgkin lymphoma, leading to a durable 18-month complete remission with no significant immediate toxicity [23]. A study comparing EAM to the BEAM regimen reported comparable 3-year OS (76% vs. 83%, *P *= 0.6) and DFS (74% vs. 83%, *P* = 0.6) [25].

Other studies have attempted to substitute carmustine with agents like thiotepa or lomustine, showing comparable efficacy with no significant differences in outcomes [36–38]. A study comparing carmustine with other alkylating agents in combination with EAM suggested a non-significantly lower rate of transplant-related death (5.5% vs. 11.4%) and similar toxicity profiles and efficacy (mean OS 52.1 vs. 18.8 months, *P* = 0.2) [36]. Our study found comparable outcomes in terms of survival and similarly demonstrated that both EAM and CEAM regimens are well tolerated and effective.

In research with similar settings, Luz et al. [39] found that survival was significantly higher in advanced stages. However, they noted that this finding could be biased due to the small cohort size. On the other hand, they demonstrated that chemosensitivity and disease status at transplantation were more important than staging in predicting outcomes. We believe this variability in results can be addressed by observing a larger number of patients or through meta-analysis of current relevant studies.

These results could be particularly useful for transplant centers with limited access to carmustine. The comparable outcomes for the EAM and CEAM regimens reinforce the notion that in this context, the choice of conditioning regimen should be guided more by disease features, such as chemosensitivity and tumor behavior. Our findings suggest that the number of previous chemotherapies and stage at diagnosis have a greater impact on survival than the conditioning regimen, indicating that patient selection and optimal transplant timing are more critical factors. In other words, patients with NHL with chemosensitivity and late relapse might benefit from a three-drug combination, avoiding the unnecessary toxicity and cost associated with more intensive regimens. This approach aligns better with disease behavior and drug availability.

It has been suggested that patients with NHL and chemosensitive, less advanced disease have better survival rates and lower NRM after ASCT. One study showed that a higher stage at diagnosis is associated with an increased relapse rate (53% vs. 34%, *P* = 0.05) [40]. Similar studies have also reported better survival and lower relapse rates in patients with less advanced disease and fewer lines of previous chemotherapy [41–43]. These findings are consistent with our results, which show the negative impact of advanced disease on OS and DFS.

In terms of statistical analysis, one of the strengths of the study is that we modeled quantitative variables (age and time to transplant) as continuous variables to preserve statistical power and avoid the loss of information associated with categorization. Several limitations, however, could affect the robustness of our results. This study was conducted on a relatively small group of patients, which limits the generalizability and strength of the evidence. The retrospective and non-randomized design of this research also introduces an inherent decrease in its statistical power. Additionally, the heterogenous nature of the lymphoma patients enrolled complicates the interpretation of the results. Despite these limitations, our study provides valuable insights into the effects of omitting carmustine in a cohort of patients with NHL.

## Conclusion

With no proven difference among conditioning regimen types, it appears that clinical condition and the availability of chemotherapeutic agents should guide the optimal choice of conditioning regimen. Our real-world data suggest that patients receiving either the EAM or CEAM conditioning regimens experience similar outcomes in terms of survival, relapse, and NRM. Both regimens were well tolerated, with a more prolonged neutropenia observed when carboplatin was added. Therefore, the omission of BCNU from the commonly used BEAM regimen appears feasible, and there may be no need to add other agents to the remaining three-drug combination.

## Data Availability

The datasets generated during and/or analyzed during the current study are available from the corresponding author on reasonable request.
